# Are Dietary Patterns Relevant for Reducing the Risk of Fractures and Sarcopenia?

**DOI:** 10.1007/s11914-024-00899-7

**Published:** 2025-01-23

**Authors:** Ailsa A. Welch, Jamie Scott, Donnie Cameron, Max Yates

**Affiliations:** 1 Norwich Medical School, University of East Anglia, Norwich, NR4 7TJ UK; 2 Centre for Population Health Research, Faculty of Medicine and Health Sciences, University of East Anglia, Norwich, UK; 3 Norwich Epidemiology Centre, Faculty of Medicine and Health Sciences, Population Health, University of East Anglia, Norwich, UK; 4 Department of Medical Imaging, Radboud University Medical Center, Nijmegen, The Netherlands; 5 Department of Rheumatology, Norfolk and Norwich University Hospital, Norwich, UK

**Keywords:** Dietary patterns, Mediterranean dietary pattern, Fractures, Sarcopenia, Bone, Skeletal muscle

## Abstract

**Purpose of Review:**

This review aims to summarise recent evidence on the effects of dietary patterns on the risk of bone fractures and sarcopenia.

**Recent Findings:**

Several dietary patterns have been investigated in relation to musculoskeletal health, including Mediterranean Dietary Patterns (MDP), Dietary Inflammatory Indices, vegetarian and vegan diets. Adherence to ‘healthier’ dietary patterns appears to be protective against fractures and sarcopenia, with the strongest protective associations found between the MDP and fractures. Individuals following vegan or vegetarian eating patterns need to be aware of calcium and vitamin D requirements to maintain musculoskeletal health.

**Summary:**

Although more healthy dietary patterns may be protective for musculoskeletal health the current evidence base is limited by variation in the construction of dietary pattern scores and reported outcome measures. Future research should fully report scoring methods, intakes of dietary components across scoring groups or categories, and consider outcome measures that allow for better comparison between studies.

## Introduction

Osteoporosis and sarcopenia are age-related diseases affecting bone and skeletal muscle, respectively. Osteoporosis results from the loss of bone microarchitecture and bone mineral density and can lead to fragility fractures, which are sustained by 1 in 3 women aged > 50 years in ‘western countries’ [1]. Sarcopenia is generally defined as the presence of low muscle mass plus low muscle strength and/or low physical function [2–8]. Despite the increasing research interest sarcopenia has received since its ICD-10-CM code was introduced in 2016 [9], no consensus has yet been reached on its precise definition or diagnosis [10]. Global prevalence is estimated to range from 10–27% [11].

Osteoporosis and associated fragility fractures increase the risk of poor physical function and disability [1, 12] and sarcopenia increases the risk of type 2 diabetes [13], frailty [14], and falls [15] (a key cause of fractures). Both diseases are associated with an increased risk of mortality [1, 12, 15]. In the US, in 2008, the cost of osteoporosis was estimated at $22 billion [16], with the annual cost of fractures predicted to rise to > $95 billion by 2040 [17]. In 2000, low muscle mass was associated with around $18.5 billion of healthcare costs [18] and, in 2014, hospitalisation costs in individuals with sarcopenia was estimated at around $40 billion [19]. Globally, between 2022 and 2050, the number of adults aged $$\:\ge\:$$65 years is predicted to more than double, from 771 million to 1.6 billion [20]. Without effective strategies for prevention, the prevalence of osteoporosis and sarcopenia—and their associated costs—is likely to rise dramatically [21, 22].

Osteoporosis and sarcopenia share many of the same age-related mechanisms, including alterations in circulating levels of hormones, inflammation, cellular senescence, and the accumulation of lipids within bone and muscle tissue (reviewed in references [23–31]). Similarly, these diseases share modifiable risk factors, including poor nutritional status [32, 33]. Specific nutrients—for example, vitamin D and calcium in relation to bone health and protein in relation to muscle health—have been extensively researched in this area. In contrast to individual nutrients, there is interest in how dietary patterns influence bone and muscle health during ageing. Dietary pattern scoring systems typically positively score intakes of healthy whole plant foods (for example fruits, vegetables, and legumes) that are high in certain micronutrients and bioactive compounds that may improve nutritional status and interact with the mechanisms of onset of osteoporosis and sarcopenia. This review discusses dietary patterns that have been investigated and evidence of their effectiveness for prevention of fragility fractures and optimising muscle health.

### Dietary Patterns

Dietary patterns are measures of the diet quality of foods and nutrients consumed.

Traditionally, nutrition epidemiology has focused on investigating associations between individual nutrients, foods, or food groups and health outcomes. In the past few decades, research has increasingly investigated whole dietary patterns, which provide a more complex summary of diet quality reflecting the combination of foods, nutrients, and bioactive compounds that are consumed, and their interactions [34, 35], which can be investigated using *a priori* or *a posteriori* methods. *A priori* methods use predefined, theoretically-driven dietary indices and scores to assess adherence to specific dietary patterns, such as the Mediterranean dietary pattern (MDP) [34]. *A posteriori* methods use statistical techniques to derive habitual dietary patterns specific to populations [34, 35]. Dietary pattern scores are calculated through assessing consumption of foods, food groups, and/or nutrients. Some also include drinks and/or dietary supplements. An overview of the dietary components that are included in common dietary patterns are provided in Tables 1 and 2.

**Table 1:**
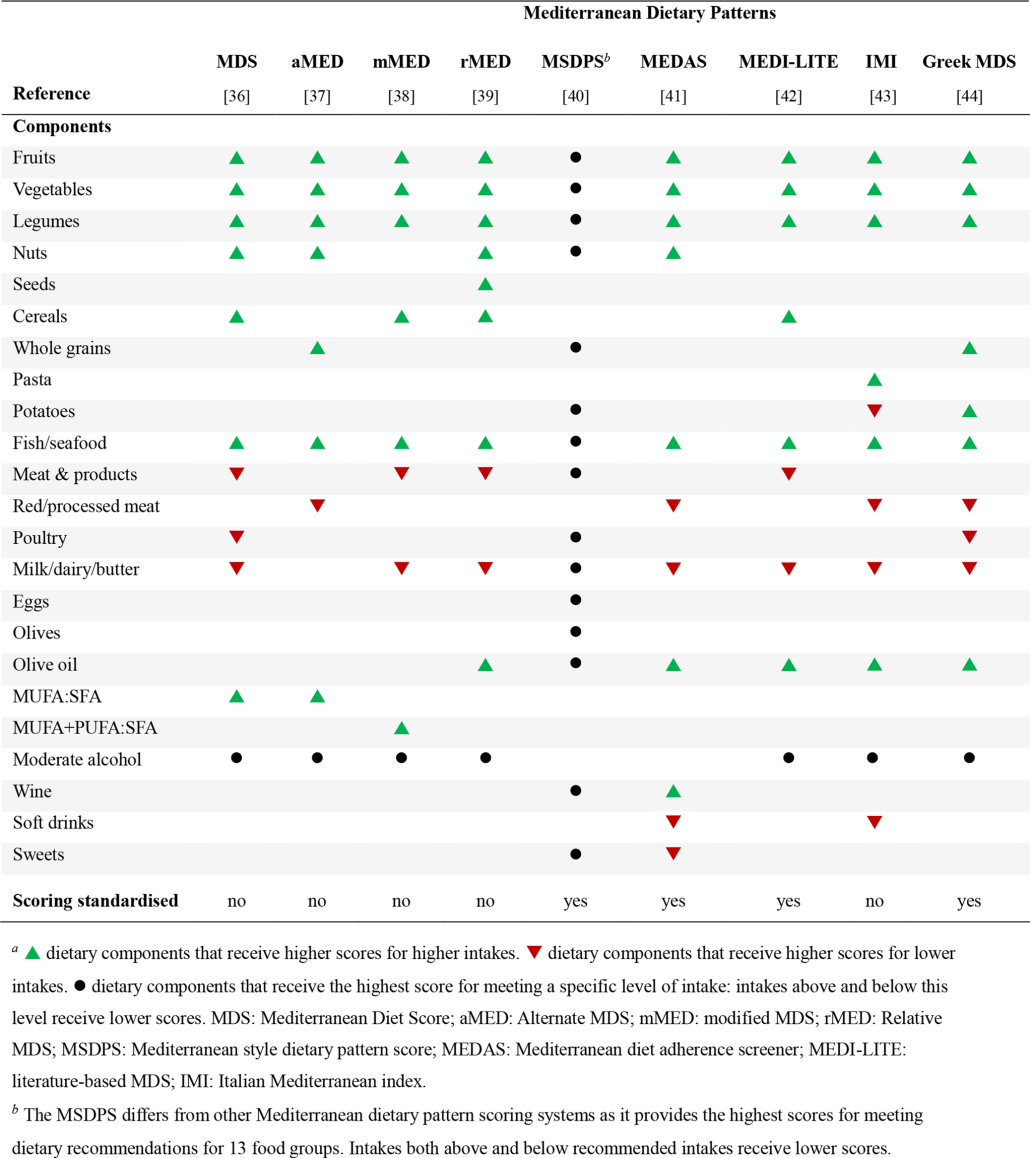
Dietary components included in a range of Mediterranean dietary pattern scoring systems, and whether standardised scoring systems are used for assessing intakes^*a*^

**Table 2:** Overview of commonly used dietary patterns, their included dietary components and use of standardised or non-standardised scoring systems^*a*^

**Dietary Patterns**	**Included Dietary Components**		**Scoring Method**
**Dietary Inflammatory Indices**	**Anti-Inflammatory Dietary Components**	**Pro-Inflammatory Dietary Components**	
**Dietary Inflammatory Index (DII)** [45]	Anti-inflammatory: Alcohol; vitamin B6; $\:\beta\:$-carotene; caffeine; eugenol; fibre; folic acid; garlic; ginger; magnesium; MUFA; niacin; n-3 fatty acids; n-6 fatty acids; onion; PUFA; riboflavin; saffron, selenium; thiamin; turmeric; vitamin A; vitamin C; vitamin D; vitamin E; zinc; green/black tea; flavan-3-ol; flavones; flavonols; flavonones; anthocyanidins; isoflavones; pepper; thyme/oregano; rosemary.	Pro-inflammatory: vitamin B12; carbohydrate; cholesterol; energy; total fat; iron; protein; saturated fat; *trans* fatty acids.	*Z*-score calculated for all dietary components using a global mean and global standard deviation (consistent between studies). Intakes multiplied by inflammatory effect scores.
Energy-Adjusted DII (E-DII) [46]	Same as DII.	Same as DII, excluding energy.	Same method as DII, with energy-adjusted intakes, global mean and global standard deviation.
Empirical Dietary Inflammatory Pattern (EDIP) [47]	Anti-inflammatory: beer; wine; tea; coffee; dark-yellow vegetables; green leafy vegetables; snacks; fruit juice; pizza.	Pro-inflammatory: processed meat; red meat; organ meat; fish (other than dark-meat fish); other vegetables (other than green leafy and dark-yellow vegetables); refined grains; high- and low-energy beverages; tomatoes.	Weighting factors provided for weighting intakes of each dietary component for EDIP score calculation.
Adapted DII (ADII) [48]	Anti-inflammatory: protein; n-3 fatty acids; fibre; ethanol; caffeine; vitamin A; $\:\beta\:$-carotene; thiamin; riboflavin; niacin; vitamin B6; folate; vitamin C; vitamin D; vitamin E; iron; magnesium; selenium; zinc; tea; quercetin; genistein; epicatechin; luteolin; daidzein; cyanidin; garlic; ginger; saffron; turmeric;	Pro-inflammatory: SFA; MUFA; *trans* fatty acids; n-6 fatty acids; cholesterol; carbohydrate; vitamin B12.	Energy-adjusted intake standardised using the population-specific mean and standard deviation. Intakes multiplied by inflammatory effect scores.
**DASH Indices**	**Higher Scores for Higher Intakes**	**Higher Scores for Lower Intakes**	
DASH (Folsom et al. 2007) [49]	Total grains; whole grains; vegetables, fruits; dairy foods; nuts, seeds and dry beans.	Meat, poultry and fish; % kcal from fat; % kcal from SFA; sweets; sodium.	Cut-off points provided for scoring.
DASH (Dixon et al. 2007) [50]	Whole grains; vegetables; fruits; dairy products; nuts seeds and legumes.	Meat and meat equivalents; saturated fat; added sugar; alcohol intake.	Cut-off points provided for scoring.
DASH (Fung et al. 2008) [51]	Fruits; vegetables; nuts and legumes; whole grains; low fat dairy.	Sodium; red and processed meats; sweetened beverages.	Score based on quintiles of intake of dietary components.
DASH (Mellen et al. 2008) [52]	Protein; fibre; magnesium; calcium; potassium.	Total fat; saturated fat; cholesterol; sodium.	Cut-off points provided for scoring.
DASH (Gunther et al. 2009) [53]	Total grains; high fibre grains; vegetables; fruits; total dairy, low fat dairy; nuts, seeds and legumes.	Meat, poultry, fish and eggs; fats and oils; sweets.	Cut-off points provided for scoring.
**Other Dietary Patterns**	**Higher Scores for Higher Intakes**	**Higher Scores for Lower Intakes**	
Plant-Based Diet Index (PDI) [54]	Whole grains; fruits; vegetables; nuts; legumes; vegetable oils; tea and coffee; fruit juices; refined grains; potatoes; sugar-sweetened beverages; sweets and desserts.	Animal fat; dairy; eggs; fish or seafood; meat; miscellaneous animal-based foods.	Score based on quintiles of intake of dietary components.
Healthful PDI [54]	Whole grains; fruits; vegetables; nuts; legumes; vegetable oils; tea and coffee.	Fruit juices; refined grains; potatoes; sugar-sweetened beverages; sweets and desserts; animal fat; dairy; eggs; fish or seafood; meat; miscellaneous animal-based foods.	Score based on quintiles of intake of dietary components.
Unhealthful PDI [54]	Fruit juices; refined grains; potatoes; sugar-sweetened beverages; sweets and desserts.	Whole grains; fruits; vegetables; nuts; legumes; vegetable oils; tea and coffee; animal fat; dairy; eggs; fish or seafood; meat; miscellaneous animal-based foods.	Score based on quintiles of intake of dietary components.
Healthy Eating Index (HEI) [55]	Total fruits; whole fruits; total vegetables; greens and beans; whole grains; dairy; total protein foods; seafood and plant proteins, ratio of PUFA plus MUFA to SFA.	Refined grains; sodium; added sugars; saturated fats.	Cut-off points provided for scoring.
Alternate HEI (AHEI) [56]	Vegetables; fruit; whole grains; nuts and legumes; long-chain n-3 fats (EPA and DHA); % kcal from PUFA; low to moderate alcohol consumption.	Sugar-sweetened beverages and fruit juice; red and processed meat; % kcal from *trans*-fatty acids; sodium.	Cut-off points provided for scoring.
Mediterranean-DASH Intervention for Neurodegenerative Delay (MIND) [57]	Green leafy vegetables; other vegetables; berries; nuts; whole grains; fish (not fried); beans; poultry (not fried); moderate wine consumption; olive oil.	Butter and margarine; cheese; red meat and products; fast fried foods; pastries and sweets.	Cut-off points provided for scoring.

^*a*^ Abbreviations: DASH: Dietary Approaches to Stop Hypertension; DHA: docosahexaenoic acid; EPA: eicosapentaenoic acid; MUFA: monounsaturated fatty acids; PUFA: polyunsaturated fatty acids; SFA: saturated fatty acids

Most dietary patterns assign positive scores to higher intakes of healthy whole plant foods, with MDPs also positively scoring consumption of fish and healthy fats (Table 1). There is more variation in the dietary components that are negatively scored (higher scores for lower intakes) between dietary patterns, with most including red and/or processed meats. Dairy products may be positively or negatively scored—sometimes dependent on the types of dairy products—or omitted completely. Dietary Inflammatory Indices differ in that they assess dietary components that are associated with inflammation.

Dietary patterns may also be assessed by inclusion or omission of specific food groups from the diet made through choice. Vegetarian diets exclude meat and may also exclude fish products, while vegan diets omit all animal foods. Vegetarian and vegan diets differ from dietary patterns since these are related to individual food choices and the ‘quality’ of the diet may vary widely within these eating patterns. As *a posteriori* patterns are specific to the population in which they are derived, this review focuses on research investigating *a priori* and choice-based dietary patterns.

## Dietary Patterns and Fracture Risk

### The Mediterranean Diet

The MDP and its relationship to fragility fractures was reviewed in a systematic and mapping review in people of all ages in 2017 [58], at which point, only two studies had investigated prospective associations between fracture risk and the Mediterranean Diet Score (MDS), which negatively scores dairy products. One further study [59] investigated the aMED (Alternative Mediterranean Diet), which does not assess dairy intake. This study, in postmenopausal US (United States) women, found no association between the aMED score and total fracture risk, but showed a 20% lower risk of hip fractures [59]. The two studies using the MDS found either no association [60] or a reduced risk of fractures [61]. One was a pan-European study [61] in 188,765 adults, with a wider range of dietary intakes in comparison with the much smaller study in France [60] (in 1,482 adults), likely affecting power to detect associations. A subsequent systematic review, in 2018, meta-analysed three studies relating the MDP to risk of hip fractures, finding a protective effect (relative risk 0.79; 95% CI 0.72–0.87) in cohorts ranging from 71,333 adults in Sweden to the 188,765 adults in the pan-European Study [61, 62]. Although the 2018 review included a further study from Sweden, the null findings from the smaller study in France [60] were not included. After these systematic reviews, three further research papers were published. The CHANCES study—in over 140,000 adults—found that individuals with either moderate or high versus low adherence to the MDS had a 7% and 6% reduced risk of hip fracture respectively [63]. In contrast, no association was found between the aMED and hip fracture risk in over 111,000 men and women from two US cohorts [64]. Most recently the UK EPIC-Norfolk Study found that higher adherence to the aMED was associated with 23% and 21% lower risk of total and hip fractures, respectively, however, the MDS was associated with reduced risk of total but not hip fractures [65]. As noted earlier, dairy foods are scored negatively in the MDS, and are omitted from the aMED, which may explain the different associations between fracture risk and these scores [66]. A study in men and women in Sweden investigated the interaction between a modified Mediterranean Diet (mMED) and calcium intake. Those with the highest calcium intakes (> 1,200 mg/d) and mMED scores were at the least risk of hip fractures [67].

Overall, three of five studies in men and four of five studies in women found protective associations between adherence to the MDP and risk of fractures.

### Vegetarian and Plant-Based Diets

Certain nutrients—notably vitamin B12, iron, zinc, and calcium—may be predominantly found in, or better absorbed from, animal or marine foods and are important for maintaining musculoskeletal health. Vegetarian or vegan diets may therefore leave individuals at risk of a shortfall of these micronutrients, predisposing these individuals to low bone density, sarcopenia, and fragility fractures. Two recent reviews have summarised the potential issues for risk of fragility fractures in those following vegetarian or vegan diets [68, 69].

Three recent population studies investigated associations between vegan or vegetarian diets and fracture risk in the UK. One—in 413,914 UK Biobank participants—found a 50% higher risk of hip fracture in vegetarians than in ‘regular meat eaters’ [70]. Lower BMI is generally more prevalent in vegans and vegetarians than meat-eaters, potentially increasing fracture risk in these groups. Interactions with BMI were found, explaining 28% of the observed risk for the differences in hip fracture between vegetarians and ‘regular meat eaters’. An earlier study comparing vegetarians and vegans with meat-eaters in the EPIC-Oxford study found the risk of hip fracture was 25% higher in vegetarians, and more than double in vegans. Vegans also sustained higher risks of total, leg, and other main site fractures than meat-eaters. These significant associations remained strong even with adjustment for BMI, dietary calcium and/or total protein [71]. A further study in 26,318 women from the UK found that vegetarians had a 33% greater risk of hip fracture compared with regular meat-eaters. Adding BMI into the analysis did not modify the associations [72].

The US Adventist Health Study 2 (in 34,542 women) found that individuals following a vegan diet sustained a 55% higher risk of hip fractures than omnivores [73], however, there was no difference in fracture risk when comparing omnivores and vegans consuming calcium and vitamin D supplements, indicating that vegans who took supplements were at no greater risk than omnivores.

Plant-based eating patterns tend to be protective for most chronic diseases; however, not all foods consumed within a plant-based diet (PBD) necessarily provide health benefits, due to the potential to consume larger quantities of ultra-processed foods. It may therefore be important to consider the quality of PBDs. Two recent studies investigated healthy versus unhealthy plant-based dietary patterns and fracture risk. The first—in 126,394 UK Biobank participants—found no associations between the risk of total, hip, or vertebral fractures and either healthful or unhealthful PBDs [74]. The second—in 70,285 US women—also found no associations between healthful or unhealthful PBDs and hip fracture when diet at baseline was assessed [75]. However, the authors found that more recent diet—assessed at the most recent data collection prior to the incident fracture, or at the end of follow up, whichever was earlier—was related to hip fractures (21% lower risk with the healthful index, 28% increased risk with the unhealthful index). The authors noted that reverse causality could not be discounted. This situation occurs when individuals change their diets according to a diagnosis of a disease. It is also possible that more recent diet is more relevant to the onset of fractures than long-term diet.

Evidence is accruing that following a vegetarian diet may increase risk of fractures, but there is limited evidence to support definitive findings in vegans. Individuals following a vegetarian or vegan diet who take supplemental vitamin D and/or calcium may be protected from this increased risk. Recent evidence also suggests that PBDs containing mostly ‘unhealthy’ plant-based foods may be relevant for fracture risk. Nevertheless, following a diet higher in healthy plant-based foods may be protective for risk of fractures.

### Dietary Inflammatory Indices

Dietary inflammatory indices assess the inflammatory potential of the diet, with higher scores reflecting a more pro-inflammatory diet. These indices have been investigated in relation to fracture risk in eight prospective cohort studies since 2016 [76–83]. The most recent investigated was the Empirical Dietary Inflammatory Pattern (EDIP) and hip fracture risk in 87,995 post-menopausal white US women [76]. After adjustment for confounders, there was a 7% increase in the risk of hip fracture per one standard deviation (SD) increase in EDIP. A further study in 1,559 US women investigated the energy-adjusted Dietary Inflammatory Index (E-DII) [77]. Each SD increment in the E-DII was associated with a 28% increased risk of total fractures. These findings were independent of contemporaneous bone mineral density measures of the femoral neck and lumbar spine.

In almost 12,000 men and women from the China Health and Nutrition Survey, there was an increased risk of fractures of 12% in women per quintile increase in the Dietary Inflammatory Index (DII), but no associations were found in men [78]. In almost 4,000 participants from the Mr./Ms.OS Hong Kong cohort, a one-unit increase in DII score was associated with a 10% higher risk of total fractures in men, but not in women [79], although there was a significant association between the DII and risk of osteoporosis in women.

In 1,098 adults from Tasmania [80] there was a 9% increase in incident total fractures for every unit increase in the E-DII score in men. However, in women, the risk of fractures decreased significantly, by 12.2% per unit increase in the E-DII, indicating a protective effect of a higher E-DII. These conflicting associations were despite both men and women experiencing substantial decreases in lumbar spine and total hip bone mineral density over 10 years. These results agree with an earlier study where postmenopausal US women with a higher E-DII score experienced a lower risk of total or lower arm fractures [81]. On the other hand, a small study from the Osteoarthritis Initiative in the US, which included 560 adults, found that a higher E-DII was associated with increasing risk of fractures in women, but not men [82]. The French *Nutri net Santé* cohort—including 15,906 adults—found no association between the Adapted DII (ADII) and risk of vertebral, major osteoporotic or low trauma fractures in men or women [83].

For the DII, four of seven studies in women found associations in the expected direction: namely, a higher DII score was related to increased risk of fractures. Of the five studies in men, two were in the expected direction, with three finding no associations. Two studies in women found a protective association with a more inflammatory DII score. These differences do not appear to be related to region; even within the US, the findings were in different directions, and in either men or women within the same cohort.

### Other Dietary Patterns

The Alternative Healthy Eating Index (AHEI) was investigated in four cohort studies, summarised in a 2018 meta-analysis [84]. Individual study sizes varied from 36,602 to 90,014 participants, with one including only women [84]. In both men and women combined, higher adherence to the AHEI was associated with a 17% reduced risk of fractures. A further sub-analysis found that the AHEI was associated with a 10% reduced hip fracture risk in women, but not men [84].

Two studies investigated associations between the DASH diet (Dietary Approaches to Stop Hypertension) and incidence of fractures [59, 64]. Haring et al. found a trend towards an association between higher DASH score and lower hip fracture risk in women from the Women’s Health Initiative Study [59]. A further study found no associations in men, but a trend towards significance across categories of the DASH score in women [64].

## Dietary Patterns and Sarcopenia

### Mediterranean Dietary Patterns

The first systematic reviews investigating MDPs and sarcopenia or its indices (muscle mass, muscle strength, and physical function measures) found too few eligible studies to reach any conclusions [58, 85]. A later systematic review and meta-analysis reported cross-sectional associations between MDPs and walking speed and knee extension strength (but not grip strength) [86], whereas the most recent, including cross-sectional and longitudinal studies, concluded that higher adherence to an MDP positively influences muscle mass and physical function, with inconclusive evidence for muscle strength [87]. Subsequent cross-sectional studies have provided mixed results, with most showing no associations with sarcopenic indices: two [88, 89] of three [88–90] found an association with muscle mass, one [91] of six [88, 91–95] found an association with muscle strength, and one [93] of three [88, 92, 93] found an association with physical function. In further longitudinal studies, adherence to an MDP was not associated with grip strength or gait speed in older men over a three-year follow-up period [96], but was associated with a slower decline in walking speed and chair-stand performance in older adults over a 12-year follow-up period [97].

Two small intervention trials have investigated the effect of an MDP on lean mass [98] and grip strength [99]. The first trial assigned 50 adults with rheumatoid arthritis (RA) to an MDP or Western diet for 10 weeks in a crossover design, finding no difference in lean mass measures following intervention with either dietary pattern [98]. The second trial assigned 106 women with RA to an MDP, exercise, or MDP plus exercise for 24 weeks. A significant improvement in grip strength was found for the exercise group only [99]. Due to the study design, it is unclear whether an MDP would have improved or maintained grip strength better than a control diet alone.

### Vegetarian and Plant-Based Diets

Chan et al. [100] systematically reviewed associations between PBDs and sarcopenia, reporting considerable heterogeneity between the 17 included studies, such that any associations remained unclear. Of the six included intervention studies, none were specifically designed to investigate the effect of a PBD on sarcopenia: two focused on weight loss, one included an intervention with fruits and vegetables, two included exercise, and one aimed to improve nutritional status in individuals with RA [100]. Only two of the 11 included observational studies investigated *a priori* or choice-based dietary patterns, whereas the remaining nine investigated other aspects of diet (e.g. individual nutrients or *a posteriori* dietary patterns). One study in around 200 Vietnamese women found no difference in lean mass when comparing individuals consuming an omnivorous or vegan diet [101]. A larger study in over 400,000 British adults found that lean mass was lower in white vegetarians and vegans compared to meat eaters, with similar results in British Indian women (but not men), however, lean mass was adjusted for age only and other potentially key confounding factors (such as physical activity) were not considered. Grip strength was also investigated and adjusted for a wider range of confounders, being lower in vegetarians compared with meat eaters in all groups except for white women [102]. Since the publication of this review [100], one further longitudinal study found that a plant-based dietary pattern was associated with lower risk of developing low muscle mass in women, but not in men [103].

An intervention trial investigating the effect of an *ad-libitum* low-fat PBD on body composition in 325 adults found that, although lean mass decreased in both the intervention and control groups, lean mass as a percentage of overall body weight increased in both groups and was significantly higher in the plant-based group. Although exercise was not included as an intervention in either group, it was encouraged, and over 80% of participants across both groups chose to exercise during the course of the study [104].

### Dietary Inflammatory Index

The association between the DII and sarcopenia was investigated in two systematic reviews and meta-analyses. Higher DII scores were associated with sarcopenia [105, 106] and lower muscle mass and strength [106], but most of the meta-analysed studies were cross-sectional. Further cross-sectional studies are in agreement with these findings: three [107–109] of four [107–110] found an inverse association between DII scores and muscle mass, one found an inverse association with muscle strength [110], and one found an association with sarcopenia diagnosis [111]. Few longitudinal studies have been conducted since the publication of the aforementioned systematic reviews, two of which used data from the same population of older Australian men and found no association between DII and grip strength or gait speed [109, 112]. However, another longitudinal study found that higher DII scores were associated a faster decline in grip strength in women, but not in men [113].

### Other Dietary Patterns

Five systematic reviews investigated associations between a wide range of dietary patterns and sarcopenia. Ramadas et al. included studies from developing economies and reported associations between higher ‘diet quality’ (reflecting healthier dietary habits) and sarcopenic indices, however, some studies showed associations in one, but not both sexes, with inconsistencies between studies [114]. Three further systematic reviews had considerable overlap in their included studies, with the first reporting weak, inconsistent, and strong evidence of associations between healthy dietary patterns and muscle mass, muscle strength and physical function respectively [115]. The second and third reviewed longitudinal studies only, concluding that current evidence was insufficient [116] or mixed [117]. The most recent systematic review and meta-analysis found an association between healthy dietary patterns and reduced risk of low gait speed, but not sarcopenia, grip strength, or other measures of physical function [118].

Subsequent studies investigating ‘other’ dietary patterns are mostly cross-sectional. A small number of longitudinal studies have mainly investigated the Healthy Eating Index (HEI) or AHEI. Higher HEI scores were positively associated with walking speed (in women, but not men) [119] and muscle quality (increased muscle density and lower intermuscular adipose tissue measured by computed tomography) [120], and higher AHEI scores were positively associated with physical performance [121]. A longitudinal study in older Japanese adults found that higher adherence to the Japanese Dietary Index—characterised by greater consumption of rice, miso, soybeans, green and yellow vegetables, and mushrooms, and less consumption of beef and pork—was associated with a lower risk of low handgrip strength. One final longitudinal study found that greater adherence to the Mediterranean-DASH Intervention for Neurodegenerative Delay (MIND) diet was associated with a slower decline in physical function and better grip strength [122].

Two small intervention trials investigated the effect of either a 12-week calorie-restricted DASH diet plus lean red meat [123] or a ‘traditional Brazilian diet’ [124] on sarcopenic indices. The first reported decreased lean mass and increased sit-to-stand performance at 12 weeks but was a single-arm trial without a control group [123]. The second included three groups, with all three groups receiving a dietary intervention. A significant change in grip strength and gait speed were reported for one group after adjusting for the change in body weight (via ANCOVA), but the results do not appear to have been adjusted for multiple testing [124].

## Discussion

A range of dietary patterns have been investigated in relation to musculoskeletal health from Mediterranean Dietary Patterns (MDP) and Dietary Inflammatory Indices to vegan and vegetarian eating choices. Adherence to ‘healthier’ eating patterns, in the main, appears to be protective against fractures and sarcopenia with the strongest protective associations observed for the MDP and risk of fractures. Although research investigating dietary patterns and fracture risk or sarcopenia has increased in recent years the evidence base is currently limited for several reasons. Many dietary pattern scores were devised in relation to cardiovascular disease risk and are therefore potentially less relevant to bone and muscle health, with the scoring of dairy foods in these indices related largely to their saturated fat content. The calcium contribution from dairy foods is high, important for bone health and, therefore, prevention of fractures. Consideration should also be given to key confounding factors that are important for musculoskeletal health (for example race and physical activity), which are not always taken into account [116, 118].

There can be substantial variation in the dietary components and methods that are used to calculate dietary scores. The MDP, for example, can be assessed through a standardised specific questionnaire [125], or calculated from data collected using other dietary assessment methods such as Food Frequency Questionnaires, from which nutrients and foods are calculated [117]. When generating dietary patterns from foods and nutrients, scoring methods may require modification if relevant dietary components were not included when dietary intakes were assessed [34]. Additionally, individual dietary components may be given binary or other categorical scores based on intake. The cut-off points used to assess intake may be standardised or provided from within a specific population. This can result in individuals from different populations with similar overall adherence scores having very different intakes of one or more dietary components [34]. Furthermore, during analysis overall adherence scores may be categorised, either into quantiles or with arbitrary cut-off points [117]. This lack of standardisation makes it difficult to compare results between different studies or to reach conclusions on the effect of a specific dietary pattern on a given health outcome. Further detail on inconsistencies with derivation and reporting of the MDP is covered in Abdelhamid et al., which investigated the variability in food and nutrient intake in studies using MDPs [126]. Few studies reported the food or nutrient composition of the MDP across the range of adherence scores or categories. Where this was reported, there was large variation in intakes across adherence scores due to inconsistencies in how the MDP is defined and how the scoring system is constructed [126]. This lack of detail and inconsistencies with derivation and reporting extends to other dietary patterns, with a systematic review including 257 studies, using a range of dietary pattern indices, finding that less than a third provided food profiles, and half did not provide nutrient profiles for dietary patterns [34]. These limitations need to be addressed in future studies so there is standardised application and reporting of dietary patterns [34, 126], with full reporting of the distribution of intakes of all dietary components used in the calculation of dietary pattern scores across groups or categories of adherence scores. This will allow for better comparison between studies, and the exploration of whether there are similarities in the underlying characteristics of a specific dietary pattern that are associated with fracture risk, sarcopenia or other health outcomes. This will also aid in the development of dietary pattern intervention strategies to be tested in future intervention trials, and in the translation of findings into public health guidelines.

Alongside variation in the methods used to assess specific dietary patterns, many of the systematic reviews discussed here report variation in the outcome measures used to assess sarcopenic indices, and in the diagnostic criteria used for sarcopenia [87, 105, 106, 114, 117]. This variation—along with the relative lack of longitudinal studies and intervention trials—stymies comparisons between studies and prevents us from coming to robust conclusions about the influence of dietary quality on sarcopenia. Future studies should consider including commonly reported sarcopenic indices as outcome measures, alongside sarcopenia diagnosis, and for incident fractures, the different types ranging from vertebral, hip, wrist and total fractures, to aid comparison between studies.

Although recommendations have been made to reduce consumption of animal-based foods and increase consumption of plant-based foods (specifically healthy unrefined plant foods) for human and planetary health [127], most of the research for fracture risk or sarcopenia focuses on choice-based vegetarian or vegan eating patterns without considering the underlying quality of these dietary patterns. Given the currently limited evidence base, further research is needed that fully reports on the quality and composition of plant-based dietary patterns, and how these relate to the risk of fractures and sarcopenia.

Currently, no randomised-controlled trials have been conducted to investigate the effect of specific dietary patterns on fracture risk, but this may be due to the high costs and time commitment required to conduct such a trial. The few trials conducted in relation to dietary patterns for sarcopenia have been small and have lacked an appropriate control group to determine the effect of diet alone.

## Conclusions

The evidence on the relationship between optimal dietary intake, measured through dietary patterns, and musculoskeletal health has grown significantly over the past decade and shows promising relevance for musculoskeletal health outcomes. However, current research is hindered by considerable variation in outcome measures, such as the types of fractures, sarcopenic indices, and the diagnostic criteria used for sarcopenia. Certain *a priori* dietary patterns, such as the MDP and the DII, also suffer from lack of consistency in their derivation, making interpretation between, and comparison across studies difficult. Overall, dietary patterns higher in healthy plant foods, particularly vegetables, and which are lower in animal sourced foods appear to be important in maintaining skeletal muscle health, and—where adequate vitamin D and calcium are provided—protecting against fragility fractures.

### Key References

- Wingrove, K., Lawrence, M. A., & McNaughton, S. A. (2022). A Systematic Review of the Methods Used to Assess and Report Dietary Patterns. *Frontiers in Nutrition*, *9*, 892,351, doi:10.3389/fnut.2022.892351.
  - Comprehensive review of the application and reporting of both *a priori* and *a posteriori* dietary patterns, highlighting the limitations that can hinder comparison of results between individual studies, particularly for informing policy decisions.
- Kraselnik, A. (2024). Risk of Bone Fracture on Vegetarian and Vegan Diets. *Curr Nutr Rep*, *13*(2), 331–339, doi:10.1007/s13668-024-00533-z.
  - Review article discussing the risk factors, and the rationale for these, for bone fractures in vegetarians and vegans.
- Abdelhamid, A., Jennings, A., Hayhoe, R. P. G., Awuzudike, V. E., & Welch, A. A. (2020). High variability of food and nutrient intake exists across the Mediterranean Dietary Pattern-A systematic review. *Food Sci Nutr*, *8*(9), 4907–4918, doi:10.1002/fsn3.1784.
  - In depth review of the variability in Mediterranean dietary pattern scoring systems, highlighting the variation in both the dietary components that are included, and the range of intakes reported across adherence scores.

## Data Availability

No datasets were generated or analysed during the current study.
